# First Fluorescence Method for Native Quantification of Pirtobrutinib Used for Treatment of Cancer in its Market Form and Biological Fluids; Application of Greenness

**DOI:** 10.1007/s10895-025-04646-8

**Published:** 2025-12-23

**Authors:** Hesham Salem, Hoda Madian, Fares Badawy, Yazed Walid, Mennatullah Kamel, Mohamed A. Sarea, Ayoub Samir, Feby Amgad, Selem Mohammed, Amany Abdelaziz

**Affiliations:** Pharmaceutical chemistry department, faculty of pharmacy, Deraya University, New Minia, Egypt

**Keywords:** Pirtobrutinib, Native fluorescence, Biological fluid, Greenness assessment

## Abstract

In order to determine the appropriate dosage of pirtobrutinib for treating mantle cell lymphoma that has returned or has not responded to prior treatment, a novel, straightforward, sensitive, and quick first spectrofluorometric approach has been devised. The technique uses 2% w/v sodium dodecyl sulfate (SDS) as an anionic surfactant to micellarly increase the drug’s natural fluorescence. Following excitation at 320 nm, the increased fluorescence intensity of pirtobrutinib was observed at 380 nm. An assay method for determining the researched drug was developed by analyzing the drug’s interaction with SDS and taking advantage of the increased fluorescence intensity. With a detection limit of 8.66 ng mL^− 1^ and a quantitation limit of 26.23 ng mL^− 1^, the relative fluorescence intensity-concentration graphs were rectilinear for the 30–1000 ng mL^− 1^ range. The results of the validation of the proposed analytical method in compliance with ICH standards were judged to be satisfactory. The suggested technique was effectively used for detection in tampered-with human plasma and pharmaceutical formulation. A number of assessment tools have lately been presented, as GAPI and AGREE.

## Introduction

Mantle cell lymphoma is treated with the anticancer medication pirtobrutinib, which is sold under the Jaypirca brand. By inhibiting Bruton’s tyrosine kinase (BTK), it prevents B cell lymphocytes from proliferating and surviving [1]. The US FDA authorized pirtobrutinib (PIR) for use in medicine in January 2023 [2] and in November 2023 by the European Union [3]. Bruises, fatigue, musculoskeletal discomfort, coughing, headaches, diarrhea, stomach pain, pneumonia, bleeding, dyspnea, nausea, edema, pyrexia, and COVID-19 are the most common side effects of this medicine when used to treat mild or chronic lymphocytic leukemia [4]. White blood cells called (B cells), a subtype of lymphocytes, are in charge of making antibodies. Abandoned B cell progression can lead to cancer. The BTK enzyme is necessary for B cells to survive and proliferate. PIR inhibits BTK in a different way than the conventional BTK inhibitor ibrutinib. It does this by preventing a genetic change (transformation at the cysteine residue C481 of BTK’s active site) that may make some tumors less sensitive to ibrutinib.

**Fig. 1:**
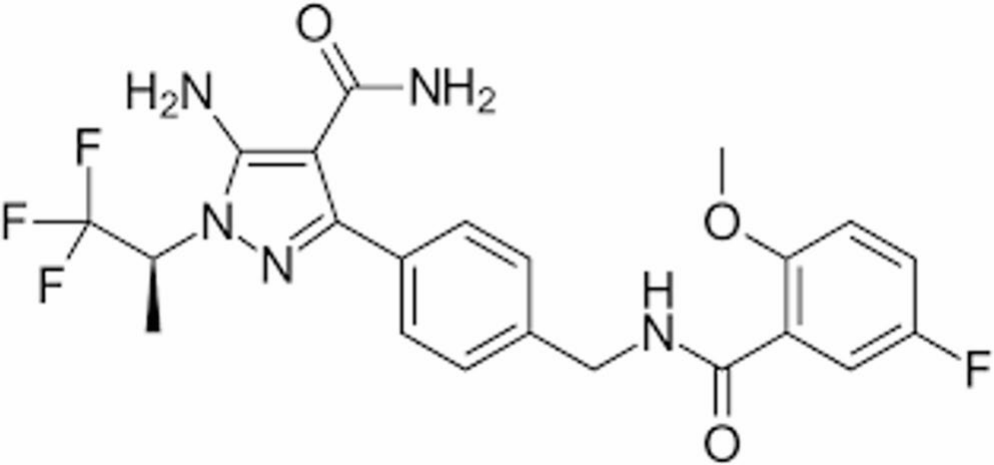
Chemical structure of PIR

In December 2023, the US Food and Drug Administration (FDA) added adults with chronic lymphocytic leukemia to the list of conditions that PIR was approved to treat [5]. The United States Food and Drug Administration authorized the use of PIR, manufactured by Eli Lilly and Company, in January 2023 to treat mantle cell lymphoma that has developed resistance to conventional BTK inhibitors [6]. A little substance called PIR inhibits BTK in a very specific non-covalent manner. Atrial fibrillation incidence has been associated with a lower chance of cessation due to unfavorable effects because of its high selectivity. Imatinib and other BTK covalent inhibitors interact with the cysteine 481 (Cys 481) amino acid in the active area of BTK; however, mutations in Cys 481 do not impact PIR ‘s inhibitory effect. Cys 481 mutations seem as the most prevalent cause of resistance to covalent BTK inhibitors, despite the fact that the precise processes underlying this resistance are yet unknown [7–9]. PIR has a low solubility in water but a high permeability in vitro. A spray-dried dispersion tablet formulation was created to provide constant oral bioavailability and lessen the variability in oral absorption [10]. As stated in the JAYPIRCA^®^ package insert, PIR’s absolute bioavailability following a single 200 mg oral dosage is 85.5% (range 75.9–90.9%). Between 0.833 and 4.15 hours, the median duration to attain maximal plasma concentration (tmax) is roughly two hours. PIR’s mean clearance is about 2.05 L/h (37.2%), and its effective half-life is roughly 19 hours. The protein binding of pirtobrutinib is about 96%, regardless of concentration, and it is mostly metabolized via CYP3A4 and directly glucuronidated by UGT1A9 and UGT1A8 in vitro. Pirtobrutinib is chemically 5-amino, as seen in Fig. 1.[(5-fluoro-2-methoxybenzoyl)amino] −3-[4-[[[methyl]phenyl]((2S)−1,1,1-trifluoropropan-2-yl) −1-[4-carboxamide pyrazole. In literature review, few chromatographic analytical techniques are available for pirtobrutinib determination [3, 11–15]. The proposed method introduces the first spectrofluorimetric method for determining PTB in biological fluid, pharmaceutical preparation, and raw materials which is developed and validated.

## Experimental

### Apparatus

A JASCO FP-83 spectrofluorimeter was used to perform the spectrofluorometric measurements. The device has a 150 W Xe-arc light and a PMT set to 400 V. The slit width of the excitation and emission monochromators was 5 nm, and their scanning speed was 1000 nm/min. Double-distilled water was produced using the Aqua¬tron water still 4000 d (Cole-Parmer, Staffordshire, UK).

The HPLC system of model acquity manufacturer Waters e 2695 ALLIANCE with quaternary pump, Rheodyne injector with 20 µL loop connected to PDA detector. The analyte was separated on symmetric shield Inertsil ODS (150 × 4.6 mm,3.5 µ) and the mobile phase containing 1.8 gm of HSA dissolved in 1 L of HPLC grade water, pH – 2.5/OPA and ACN in the ratio 60:40% v/v. The flow rate was maintained as 1mL/min and injection volume was 10 µL. The run time was set as 5 min. HPLC grade water and acetonitrile (ACN, HPLC grade) were used throughout the analysis.

### Materials and Reagents

PIR with > 99% purity was acquired from DC Chemicals, a Shanghai, China-based corporation. SDS purchased El Nasr Chemical Co. (Abo-Zaabal, Cairo, Egypt). Phosphoric acid, citric acid, boric acid, acetic acid, sodium acetate, hydrochloric acid, sodium hydroxide, carboxymethyl cellulose, cyclodextrin, ethanol, methanol, acetonitrile, diethylformamide, and Tween 60 and 80. Different buffer solution types with different pH ranges were made using distilled water; To prepare an acetate buffer, dissolve 34 g of sodium acetate in water, then add 10 mL acetic acid to adjust the pH to the desired level, and finally dilute the solution to the final volume 250 mL with distilled water. To prepare a Teorell buffer (also known as Teorell and Stenhagen buffer), you mix appropriate volumes of 1 M citric acid, 1 M phosphoric acid, and 1 M sodium hydroxide, then adjust the pH to the desired level using 0.1 M hydrochloric acid. This universal buffer can cover a broad pH range from 2 to 12. For phosphate buffer, begin with 800 mL of distilled water, measure out and add 8 g of NaCl, add 1.44 g of Na_2_HPO_4_, 0.2 g of KCl, 0.24 g of KH_2_PO_4_ Bring the pH to 7.4 or 7.2 (depending on the application) using HCl. Add distilled water until the total volume reaches 1 L. Store at room temperature.

The exact amounts of each component depend on the desired molarity and pH, so it’s crucial to follow a specific recipe for your needs.

Human plasma were given with grace by the Minia University Hospital in Minia, Egypt. They were then carefully thawed and kept at −20 °C until the test. Pharmaceutical versions of pirtobrutinib, as the Jaypirca^®^ tablets of 50 mg, produced by Eli Lilly & Company, were purchased from local markets.

### Preparations of the Standard Drug Solution

To make stock solutions (100 µg mL^− 1^) of PIR, a precisely weighed PIR powder equal to 10 mg PIR was put into volumetric flask of a 100 mL, diluted with distilled water, and completely dissolved before being topped off with distilled water. To obtain the working solutions for PIR, further dilutions with distilled water were made just before use. To make an SDS 10 mM solution, a precise 290 mg of SDS powder was introduced to a 100 mL volumetric flask, diluted with distilled water, and completely dissolved before being topped off with distilled water.

### General Analytical Procedure

Sets of volumetric flasks of 10.0 mL should be filled with precisely measured aliquots of the working solutions so that the final concentration for PIR ranges from 30 to 1000 ng mL^− 1^. Acetate buffer pH 6 and 1 ml of SDS (10 mM) solution should then be added to each flask, and the volume should be topped off with distilled water to reach the 10-ml threshold. A portion of the solution was translocated to the cuvette, and the fluorescence intensity was measured for PIR at λ_ex_ = 320 nm and λ_em_ = 380 nm. The slit width for the emission and excitation monochromators was set at 5 nm. Each measurement was made at room temperature in a 1 cm quartz cell.

### Procedure for Pharmaceutical Dosage Form

Twenty Jaypirca^®^ tablets were precisely weighed, ground into a fine powder, and thoroughly combined. A precise quantity equal to 10 milligrams of PIR was weighed and put into a 100 milliliter volumetric flask, where it was dissolved in roughly 50 milliliters of distilled water. The flask’s contents were swirled, 10 min of sonication, and then filled to the volume with distilled water. Following a thoroughly mixing and filtering the materials, the filtrate’s first part was thrown away. Following the usual analytical technique, the produced solution was quantitatively further diluted to achieve a final concentration within the calibration’s concentration range.

### Procedure for Spiked Human Plasma

In accordance with institutional protocols, the plasma was collected from male, healthy, normal volunteers at Minia University Hospital in Minia, Egypt. A heparinized tube containing 5.0 mL of drug-free human blood was vortexed for 60 s at 2000 rpm and centrifuged at 4000 rpm for 20 min. The volume of plasma used was 0.1 mL. This was specified in the method’s description, which stated that the plasma was prepared using protein precipitation with 0.1 mL of plasma for a simple and efficient sample preparation process.

One milliliter of stock solution was added to 1.0 milliliters of the drug-free plasma (supernatant) in a 10-milliliter stoppered calibrated tube. As a protein precipitating agent, two milliliters of acetonitrile were diluted via distilled water to the appropriate volume. Centrifuged for approximately 15 min at 4000 rpm. Acetonitrile is crucial for drug analysis in plasma because it is an excellent solvent for both polar and non-polar compounds Its properties facilitate accurate and efficient separation and detection of drugs, while its use in deproteinization helps remove interfering proteins from plasma samples. To obtain solutions that fall within the range of concentration the medications under study, a specific volume of the supernatant (300, 600 and 900 ng ml^− 1^) was translocated to a series of volumetric flasks of 10 ml. The general analytical technique was then carried out. The drug-free blood sample was treated in the same way without the drug in order to conduct a blank experiment.

## Results and Discussion

**Fig. 2:**
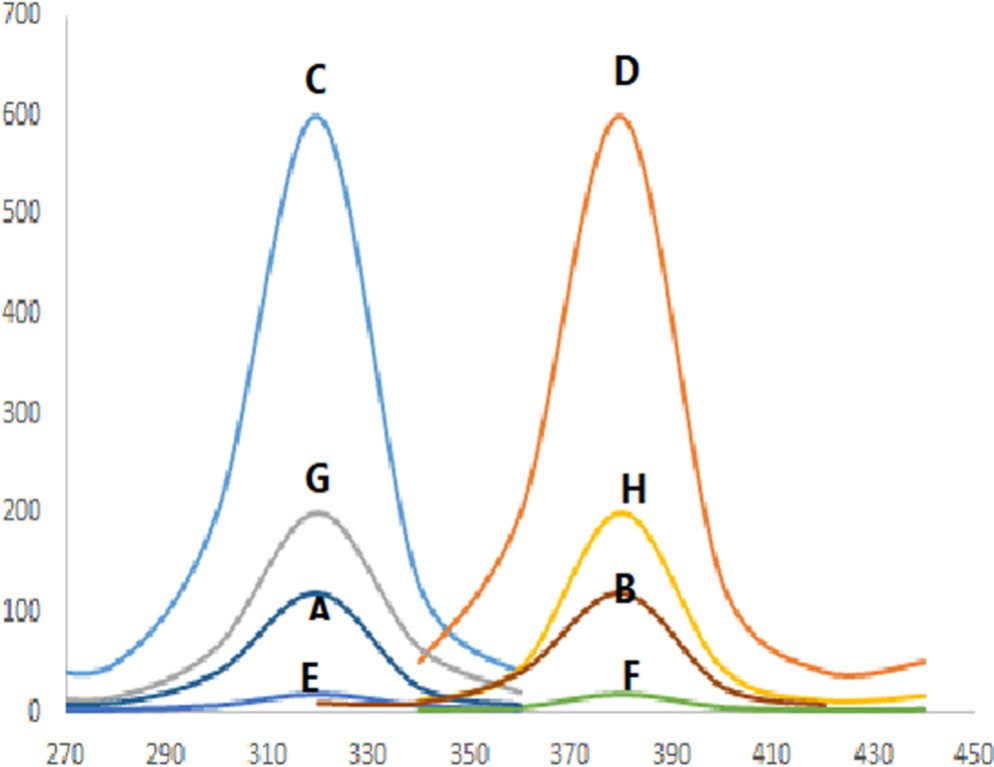
The fluorescence spectra of PIR (600 ng mL^− 1^), where (**A**) and (**B**) are excitation and emission spectra of aqueous PIR solution alone, respectively and (**C**) and (**D**) are excitation and emission spectra of PIR in presence of 2% w/v SDS, respectively, (**E**) and (**F**) are plasma blank and (**G**) and (**H**) are excitation and emission spectra of PIR in presence of spiked plasma

This study established a new spectrofluorometric approach for PIR analysis that is easy to use, sensitive, economical, and environmentally friendly. This analytical technique relies on the enhancement of the drug’s native fluorescence in the presence of sodium dodecyl sulfate (SDS). Micelle formation between the drug under study and the anionic surfactant SDS results in a 200% increase in native fluorescence (Fig. 2). Also, Fig. 2 showed the fluorescence spectra of PIR solution alone (600 ng mL^− 1^), excitation and emission spectra of PIR in presence of 2% w/v SDS, plasma blank and excitation and emission spectra of PIR in presence of spiked plasma.

### Optimization of Variables

The formation and stability of the micelle between the medication under study and SDS are influenced by many experimental conditions. While the others remained constant, each of these parameters was altered separately. SDS volume, reaction duration, dilution solvent, buffer type and pH, and the impact of an additional surfactant were among the variables examined.

#### Effect of Buffer Type and pH

**Fig. 3:**
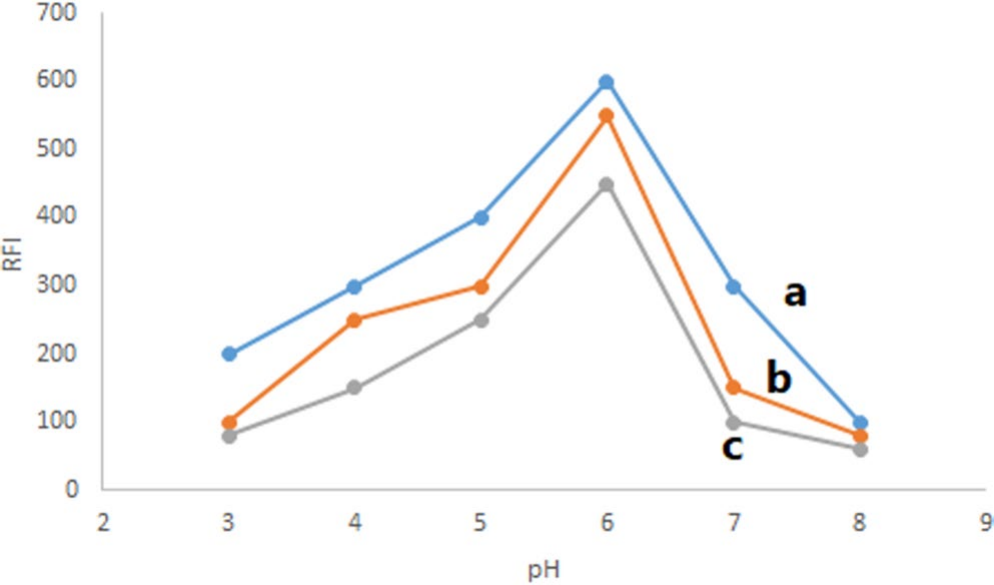
Effect of buffers (acetate buffer (**a**), Torell buffer (**b**) and Phosphate buffer (**c**)) and pH on RFI of PIR, 600 ng mL^− 1^

Various buffer types with varying pH ranges were investigated to determine the best kind of buffer and pH for micelle production. As Fig. 3 illustrates, acetate buffer at pH 6 was determined to be the most appropriate for the fluorescence amplification of PIR in micellar medium with SDS. This buffer yields a larger RFI than both phosphate buffer and Theorell and Stenhang buffer at the same pH. Signals decrease at pH values higher than 6 because the buffer’s effective range may be exceeded, leading to a decrease in buffering capacity and an inability to maintain a stable pH, which can negatively affect the drug being studied. Additionally, high pH conditions can cause alter chemical reaction equilibria, or negatively affect the stability of the studied drug.

#### Effect of Surfactants and Effect of SDS Volume

**Fig. 4:**
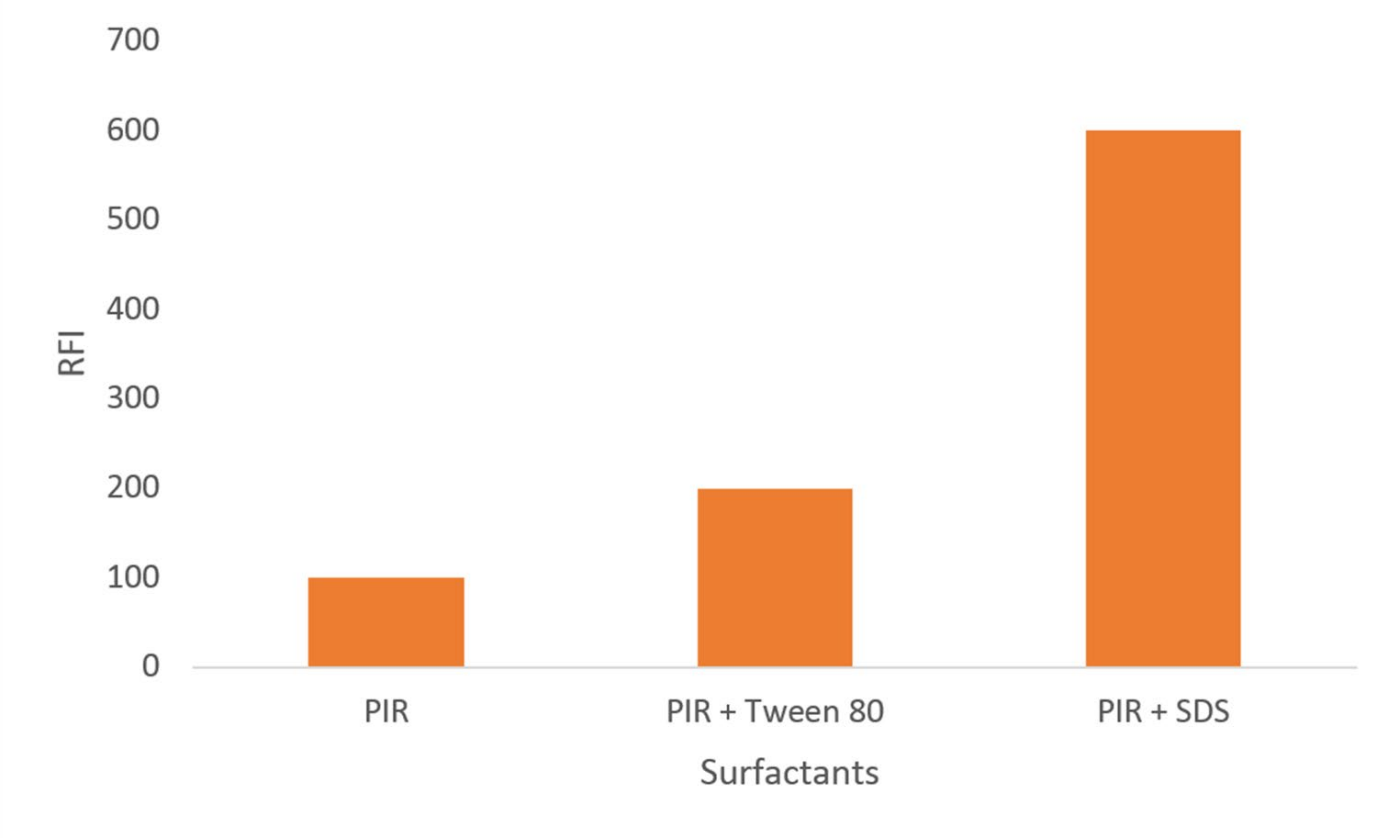
Effect of the presence of surfactants on the native fluorescence of PIR, 600 ng mL^− 1^

Utilizing numerous quantities of two various surfactants as Tween 80 & SDS in concentration 2% at various values of pH, the influence of additional chemicals to the quantification medium on the fluorescence’s native of the stated drug was studied. The utilizing of SDS observed about 200% enhancement on the RFI levels, while the utilizing of Tween 80, RFI was significantly reduced as a result of this (Fig. 4).

**Fig. 5:**
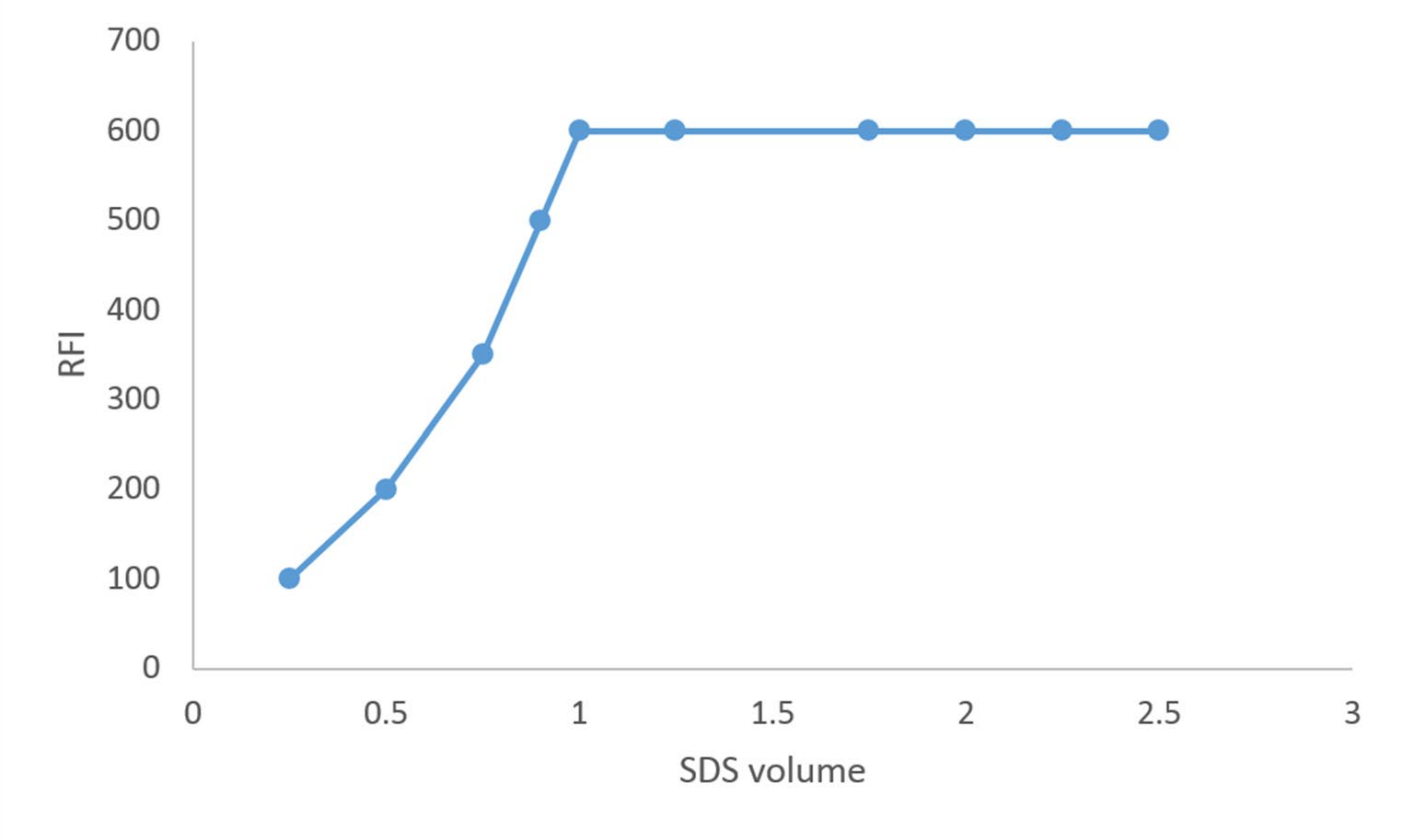
Effect of SDS volume of RFI of PIR, 600 ng mL^− 1^

Various volumes of SDS (10 mM) were obtained in order to determine the ideal SDS volume for the micelle production. As the volume of SDS was increased to 1 ml, it was found that the fluorescence intensity increased gradually. Following that, a negligible rise in fluorescence intensity was noted (Fig. 5). So, for the general analytical technique, one milliliter of SDS (10 mM) was selected.

#### Effect of Diluting Solvent

**Fig. 6:**
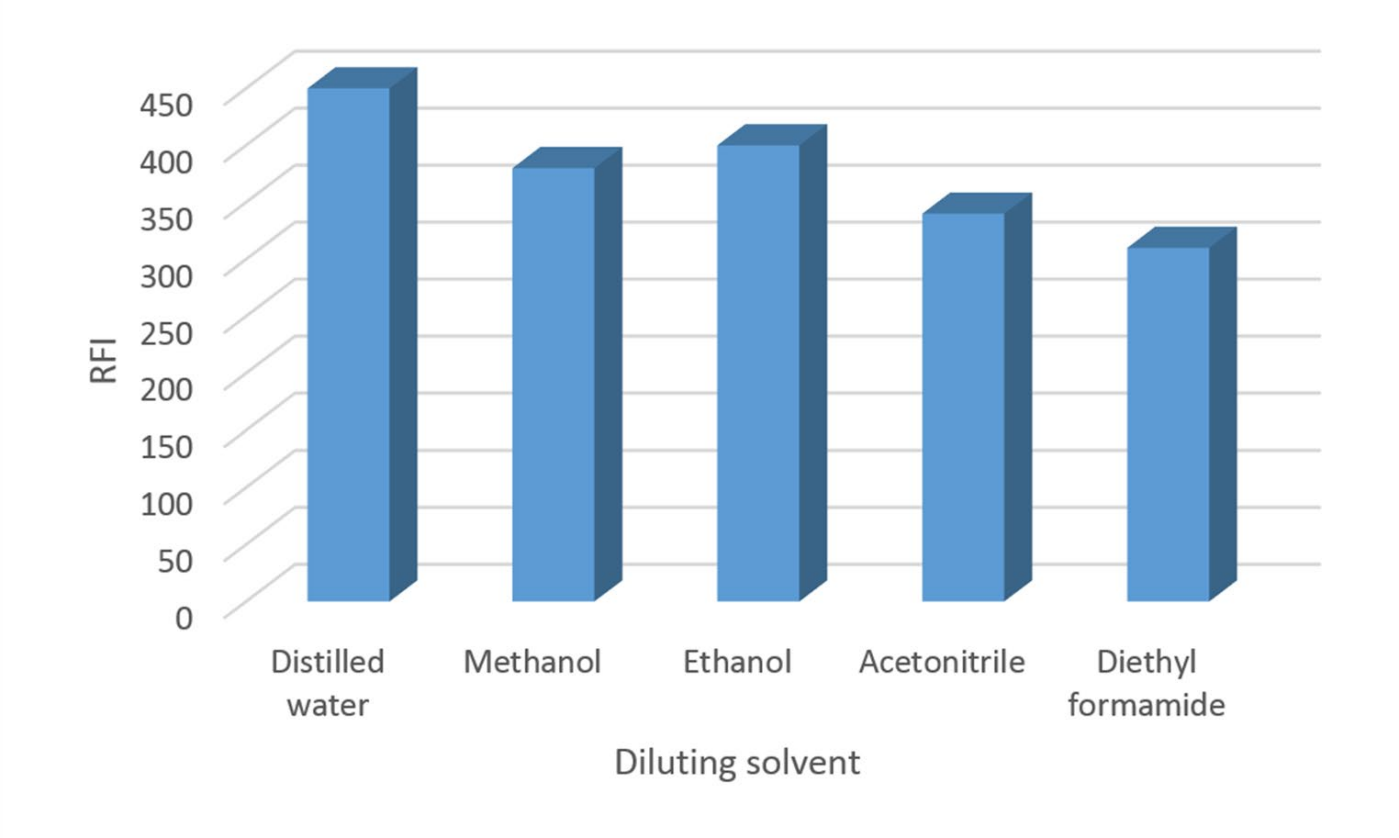
Effect of diluting solvent on RFI of PIR, 600 ng mL^− 1^

A variety of solvents, including distilled water, methanol, ethanol, acetonitrile, and dimethyl formamide, were assessed in order to determine which one would be best for dilution. Among the investigated solvents, distilled water had the highest RFI value, making it the ideal solvent for dilution (Fig. 6). For the general analytical technique, distilled water was chosen as the dilution solvent.

The decreasing effect on analytical signals from methanol and acetonitrile can be explained by their stronger elution strength and higher polarity compared to water, which can cause analytes to elute faster and reduce their response time or concentration in the detection zone. Additionally, the solvents can cause **a** decrease in analyte solubility in the matrix.

#### Effect of Time

**Fig. 7:**
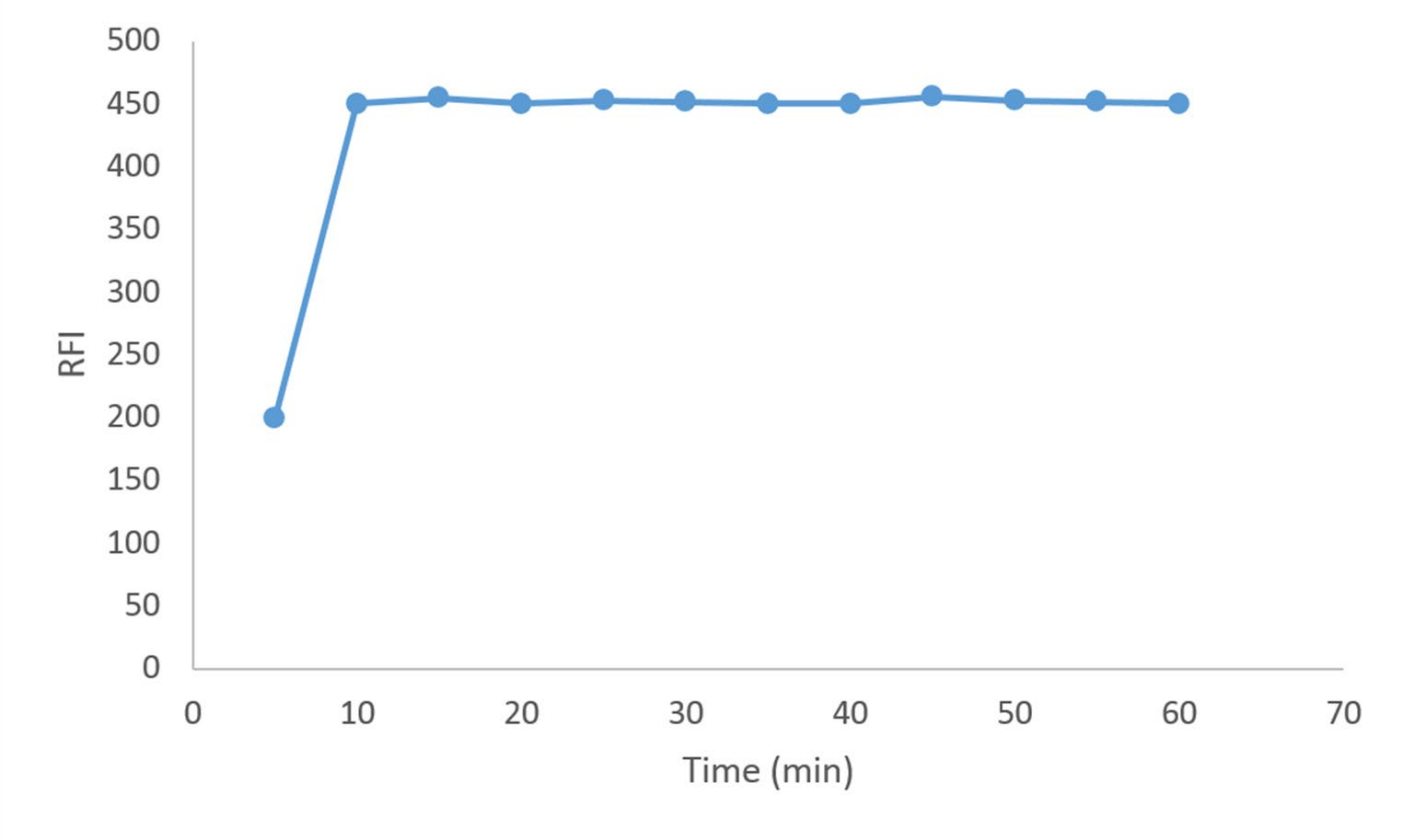
Effect of time on RFI of PIR, 600 ng mL^− 1^

The fluorescence intensity was continuously recorded for 60 min in order to determine the ideal time for micelle production. After ten minutes, the fluorescence intensity reached its maximum and remained steady for sixty minutes. Figure 7. Thus, ten minutes after the reactants were mixed, the fluorescence intensity was measured.

### Validation of the Proposed Method

In terms of linearity, accuracy, precision, robustness, selectivity, limit of detection (LOD), and limit of quantification (LOQ), the suggested analytical method was validated in compliance with ICH criteria Table 1 [16].

**Table 1:** Analytical parameters for determination of PIR by the proposed spectrofluorometric method

Parameters	
Λ_ex_ (nm)	320
Λ_em_ (nm)	380
Linearity range (ng mL^− 1^)	30–1000
Correlation coefficient (r)	0.9999
Determination coefficient (r^2^)	0.9999
Linearity range (ng mL^− 1^)	30–1000
Slope	0.9927
SD of slope (Sb)	0.0042
Intercept	5.4128
SD of intercept (Sa)	2.604
LOD (Limits of Detection) (ng mL^− 1^)	8.66
LOQ (Limits of Quantitation) (ng mL^− 1^)	26.23

#### Accuracy and Precision

The accuracy of the proposed fluorometric method was evaluated using five concentration levels within the specified PIR range. For every concentration, three copies were created. The mean of the three measurements was calculated as follows. The measurement findings were shown as a percentage recovery ± standard deviation in Table 2. The obtained results show a strong agreement between the measured and true values, indicating the good accuracy of the proposed method. To evaluate intra-day precision, three PIR concentrations were examined three times in quick succession. To evaluate the inter-day precision, three PIR concentrations were reproduced across three days in a row. The intra-day and inter-day precision results are summarized in Table 3. At both repeatability and moderate precision levels, the computed relative standard deviations of various measurements were less than 2%, demonstrating the good precision of the suggested approach.

**Table 2.** Evaluation of the accuracy of the proposed analytical procedure for determination of PIR with SDS at five concentration levels within the specified range

Sample number	Taken (ng mL^−1^)	Found ^a^ (ng mL^−1^)	% recovery	%RSD
1	30	29.80	99.32	1.03
2	100	100.58	100.58	0.75
3	300	300.33	100.11	0.95
4	600	602.22	100.37	0.77
5	900	908.91	100.99	1.11

*SD* Standard deviation, *RSD *relative standard deviation; mean of three replicate measurements

**Table 3.** Evaluation of the intraday and interday precision of the proposed spectrofluorometric method for determination of PIR in pure form

Precision level	Concentration (ng mL^− 1^)	% Recovery ± SD	RSD
Intraday	300	99.65 ± 1.45	1.46
	600	100.75 ± 1.00	0.99
	900	100.36 ± 0.94	0.94
Interday	300	100.11 ± 0.84	0.84
	600	99.05 ± 1.48	1.49
	900	100.30 ± 0.78	0.78

#### Limit of Detection (LOD) and Limit of Quantitation (LOQ)

Using the formulas LOD = 3.3 σ/S and LOQ = 10 σ/S, where S is the calibration curve’s slope and σ is the standard deviation of the intercept, the limits of quantification (LOQ) and limits of detection (LOD) were calculated based on the standard deviation of response and the slope of the calibration curve. Table 1 displayed the results that were obtained. The quantification limit was 26.23 ng mL^− 1^. When compared to the published chromatographic method, this suggests that the proposed spectrofluorometric approach has a high sensitivity.

#### Robustness

The ability of an analytical process to withstand minor but intentional changes in technique parameters is known as its robustness. One experimental variable was changed while holding the others constant in order to evaluate the robustness of the suggested spectrofluorimetric approach. Time, SDS volume, and buffer solution pH were among the variables under investigation. The performance of the recommended process was not significantly impacted by slight changes in any of these factors, according to the data shown in Table 4. This demonstrated the suggested method’s dependability.

**Table 4:** Robustness study of the proposed spectrofluorometric method for determination of PIR (600 Ng mL^− 1^) in pure form

Variation	% Recovery ± SD
Effect of pH (acetate buffer)	
pH 5.5	98.87 ± 0.86
pH = 6	100.86 ± 0.78
pH = 6.5	99.53 ± 0.58
SDS volume	
0.75 mL	98.39 ± 0.89
1.0 mL	100.86 ± 0.57
1.25 mL	100.96 ± 0.66
Effect of time	
5 min	99.00 ± 0.68
10 min	99.89 ± 0.87
15 min	99.33 ± 0.88

### Application

#### Application to Pharmaceutical Dosage Forms

JAYPIRCA^®^^(^Pirtobrutinib tablets) are supplied as 50 mg or 100 mg film- coated tablets for oral administration. Each JAYPIRCA^®^ tablet contains croscarmellose sodium, hypromellose acetate succinate, lactose monohydrate, magnesium stearate, microcrystalline cellulose and silicon dioxide.

PIR in its medicinal dose form was successfully determined using the suggested approach. By monitoring any interference from tablet excipients, the method’s selectivity was examined. It was demonstrated that the suggested approach is unaffected by tablet excipients. Student’s t-test and F-test were 0.98 and 1.99, respectively, used to compare the accuracy and precision of the results from this suggested approach with those from the stated method. Since the computed values did not surpass the theoretical values at the 95% confidence level, it evident that there is no significant difference between the findings from the suggested approach and the reported method [13], as shown by the Student’s t-test and F-test. This suggests that the proposed approach has a high degree of accuracy and precision.

#### Application to Spiked Human Plasma

The suggested analytical technique was effectively used to identify the PIR medication in human plasma that had been tampered with. The corresponding regression equation was used to calculate the drug’s concentration. The plasma was spiked with PIR standard solutions to achieve final concentrations of 300, 600, and 900 ng mL^− 1^. The outcomes were displayed in Table 5. Three drug concentrations in plasma were reported to have mean percent recoveries ranging from 96.36 to 97.99, with standard deviations ranging from 1.06 to 1.93. This suggests that the medications under study can be successfully identified in human plasma that has been spiked with a high level of precision and accuracy free from interference. These findings imply that the suggested analytical technique may be able to ascertain the drug’s concentration in actual human plasma samples following oral administration without experiencing appreciable matrix-related interference.

**Table 5:** Application of the proposed spectrofluorometric method for determination of PIR in spiked human plasma

Added concentration ng mL^− 1^	Found concentration^a^ ng mL^− 1^	% Recovery ± SD
300	289.08	96.36 ± 1.75
600	584.58	97.43 ± 1.93
900	881.91	97.99 ± 1.06

^a^ Mean of five determinations

### Assessment of the Greenness of the Proposed Method

In order to evaluate the ecological implications of the analytical approaches, a number of assessment tools have lately been presented. The evaluation of analytical techniques aids in lowering the pollution that these activities produce in the environment. For example, traditional HPLC systems can produce 0.5 L of organic waste per day on average. 2018 saw the launch of the Green Analytical Procedure Index (GAPI) [17]. Each of the 15 pictograms that GAPI offers represents a step in the five primary pentagrams that correlate to an analytical procedure. Red, yellow, and green are indicated by the color code used in GAPI. The ecological impacts that are most and least significant are indicated by the colors red and green, respectively. Only two red pictograms are displayed in GAPI assessment for pharmaceutical dosage form analysis. The sample handling pentagram, which represents the off-line sampling, displays the two red pictograms. Regulations requiring segregation between pharmaceutical production and/or clinical observation locations and quality control (QC) laboratories lead to off-line sampling, which in turn necessitates sample transportation. Even though pharmaceutical dosage forms don’t need to be preserved specifically, if the suggested approach were used on a plasma sample, three red pictograms would appear, two of which would correspond to the dosage forms. The sample preparation pentagram, which shows the use of a small volume of acetonitrile (ACN), a non-green solvent, contains the third red pictogram. Regarding equipment, chemicals, or trash produced, the other primary stages outlined in the suggested approach are environmentally safe and sustainable. Another recently released assessment tool based on the color code based on GAPI is called AGREE [18]. It was founded on the twelve green analytical chemistry (GAC) principles, which is the primary distinction from GAPI. AGREE displays a clock-shaped symbol with 12 pieces along its perimeter, each of which represents a GAC principle. A numerical value indicating the ecological impact is displayed in the pictogram’s center; the closer the value is to 1, the greater the impact. Table 6 illustrates that AGREE has a modest ecological impact, as indicated by the numerical values of 0.92 and 0.86 for dosage form and plasma samples, respectively. With the exception of the third GAC principle, which deals with off-line sampling and is unavoidable, as explained in the GAPI pictogram discussion, the perimeter is nearly greener. The use of ACN in plasma samples for protein precipitation prior to analysis is the cause of the minor ecological impact observed in the AGREE assessment’s tenth, eleventh, and twelfth principles. Since no organic solvents are needed, the suggested procedure would be completely environmentally friendly if it were used for pharmaceutical dosage form analysis. The suggested methodology’s improved environmental friendliness can be attributed to its increased throughput, low energy spectrofluorometric equipment, and straightforward sample preparation steps that eliminate the need for derivatizing agents.

**Table 6.**
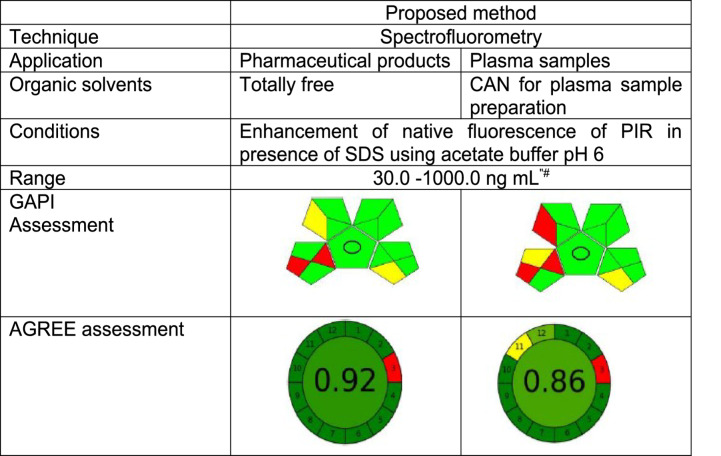
Penalty points calculation for the greenness evaluation of the present method

## Conclusion

The proposed technique is simple, economical, extremely sensitive, rapid, less time-consuming, and doesn’t require any PIR preparation prior to analysis. Since the current study does not require time-consuming liquid-liquid extraction or rely on pricey or necessary chemical reagents or expensive equipment, it is more straightforward and cost-effective. Due to the time and cost savings, these advantages also enable the recommended approach to be applied for routine quality control examination of these drugs. Additionally, the proposed method is capable of measuring the material in nanograms per milliliter. As a result, it can be used to detect the drugs being investigated in real human plasma samples.

## Data Availability

All data underlying the results are available as part of the article and no additional source data are required.
